# Unfolding is the driving force for mitochondrial import and degradation of the Parkinson's disease-related protein DJ-1

**DOI:** 10.1242/jcs.258653

**Published:** 2021-11-25

**Authors:** Bruno Barros Queliconi, Waka Kojima, Mayumi Kimura, Kenichiro Imai, Chisato Udagawa, Chie Motono, Takatsugu Hirokawa, Shinya Tashiro, Jose M. M. Caaveiro, Kouhei Tsumoto, Koji Yamano, Keiji Tanaka, Noriyuki Matsuda

**Affiliations:** 1 Ubiquitin Project, Tokyo Metropolitan Institute of Medical Science, 2-1-6 Kamikitazawa, Setagaya, Tokyo 156-8506, Japan; 2 Laboratory of Protein Metabolism, Tokyo Metropolitan Institute of Medical Science, 2-1-6 Kamikitazawa, Setagaya, Tokyo 156-8506, Japan; 3 Cellular and Molecular Biotechnology Research Institute, National Institute of Advanced Industrial Science and Technology (AIST), Tokyo, Japan, 2-4-7 Aomi, Koto-ku, Tokyo 135-0064, Japan; 4 Computational Bio Big-Data Open Innovation Laboratory (CBBD-OIL), AIST, Waseda University, 3-4-1 Okubo, Shinjuku-ku, Tokyo 169-8555, Japan; 5 Division of Biomedical Science, Faculty of Medicine, University of Tsukuba, 1-1-1 Tennodai, Tsukuba, Ibaraki 305-8575, Japan; 6 Transborder Medical Research Center, University of Tsukuba, 1-1-1 Tennodai, Tsukuba, Ibaraki 305-8575, Japan; 7 Department of Material and Biological Chemistry, Faculty of Science, Yamagata University, Yamagata 990-8560, Japan; 8 Laboratory of Global Healthcare, Graduate School of Pharmaceutical Sciences, Kyushu University, Maidashi 3-1-1, Higashi-ku, Fukuoka 812-8582, Japan; 9 Department of Bioengineering, Graduate School of Engineering, The University of Tokyo, 7-3-1 Hongo, Bunkyo-ku, Tokyo 113-8656, Japan

**Keywords:** Mitochondria, Protein import, Parkinson's disease, DJ-1, *PARK7*

## Abstract

Diverse genes associated with familial Parkinson's disease (familial Parkinsonism) have been implicated in mitochondrial quality control. One such gene, *PARK7* encodes the protein DJ-1, pathogenic mutations of which trigger its translocation from the cytosol to the mitochondrial matrix. The translocation of steady-state cytosolic proteins like DJ-1 to the mitochondrial matrix upon missense mutations is rare, and the underlying mechanism remains to be elucidated. Here, we show that the protein unfolding associated with various DJ-1 mutations drives its import into the mitochondrial matrix. Increasing the structural stability of these DJ-1 mutants restores cytosolic localization. Mechanistically, we show that a reduction in the structural stability of DJ-1 exposes a cryptic N-terminal mitochondrial-targeting signal (MTS), including Leu10, which promotes DJ-1 import into the mitochondrial matrix for subsequent degradation. Our work describes a novel cellular mechanism for targeting a destabilized cytosolic protein to the mitochondria for degradation.

## Abstract (precis)

**Summary:** Several mutations in Parkinson's disease-related protein DJ-1 cause its mitochondrial import and degradation. We reveal that protein unfolding is the driving force for the import and degradation of DJ-1.

## INTRODUCTION

A close relationship between neurodegenerative disease and impaired mitochondrial function has been widely recognized. Parkinson's disease is the best-elucidated example, with several genes associated with familial early-onset Parkinsonism implicated in mitochondrial quality control. One of those genes, *PARK7*, encodes the cytosolic protein DJ-1. DJ-1 is a relatively small (189 amino acids; <20 kDa) multifunctional protein. DJ-1/*PARK7* was first identified as an oncogene (Nagakubo et al., 1997), but was later re-identified as causal for recessive familial Parkinsonism, *PARK7* (Bonifati et al., 2003). Countless studies focused on elucidating DJ-1 functionality have since accentuated the pleiotropic nature of the protein. Although conclusions from the myriad studies vary, and frequently contradict one another, links between mitochondrial integrity and DJ-1 have been frequently observed.

The mitochondrial localization of DJ-1, however, is controversial as wild-type (WT) and mutant DJ-1 have been reported to localize to the cytosol or the nucleus (Bjorkblom et al., 2014; Blackinton et al., 2009; Cali et al., 2015; Canet-Avilés et al., 2004; Nural et al., 2009; Ren et al., 2012; Xu et al., 2005; Zhang et al., 2005). Consistent with an initial report (Maita et al., 2013), an earlier study by our group found that WT DJ-1 is cytosolic under steady-state conditions and that pathogenic mutations in the protein trigger translocation to the mitochondrial matrix through an unknown pathway (Kojima et al., 2016). In general, missense mutations in cytosolic proteins rarely result in translocation to the mitochondria. We thus wanted to disentangle the molecular mechanisms underlying the mitochondrial localization of DJ-1 mutants, and then determine the physiological significance of the mitochondrial import.

Here, we show that mitochondria-localized mutants of DJ-1 are prone to unfolding. We found that a reduction in DJ-1 structural stability promotes import into the mitochondrial matrix and subsequent degradation. Furthermore, enhancing the structural stability of DJ-1 can recover the cytosolic localization, implying that protein unfolding is the motive force for DJ-1 mitochondrial import. We thus propose a new mechanism by which destabilized cytosolic proteins (e.g. DJ-1) can be targeted to mitochondria.

## RESULTS

### Screening for novel DJ-1 mutations that promote mitochondrial localization

Several researchers, including our group, have independently reported that some pathogenic DJ-1 mutants (M26I, E163K and L166P) localize to the mitochondria (Bonifati et al., 2003; Kojima et al., 2016; Maita et al., 2013). Furthermore, proteinase K protection assays of mitochondria isolated from cells expressing the DJ-1 mutants confirmed that the DJ-1 E163K and L166P mutants localize in the mitochondrial matrix despite the absence of a predictable mitochondria/matrix-targeting signal (MTS) (Kojima et al., 2016). We thus sought to elucidate the molecular mechanism underlying the mitochondrial import of DJ-1.

**Fig. 1.**
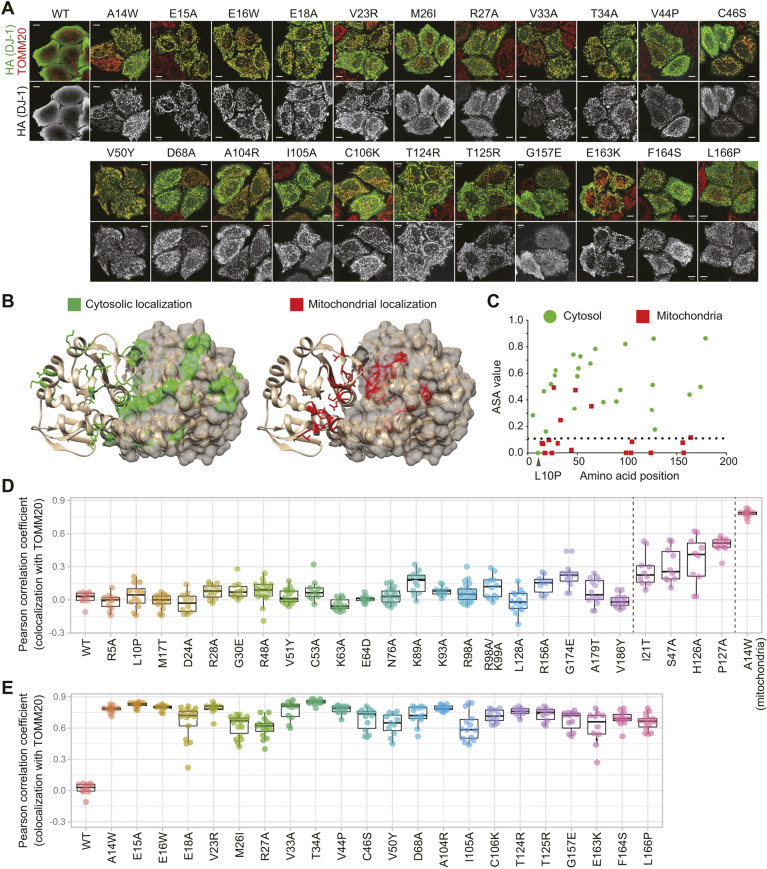
**Mutations buried in the DJ-1 structure responsible for mitochondrial localization.** (A) Mitochondrial localization of newly identified DJ-1 mutants. Top images, merged images for the DJ-1 construct signal (HA, green) and the mitochondrial marker TOMM20 (red) in DJ-1-knockout cells; bottom images, DJ-1 construct signal alone. Representative images of two independent experiments are shown. Scale bars: 10 µm. (B) Location of the mutation sites in relation to the DJ-1 dimer structure are shown. One DJ-1 monomer is shown in ribbon format, whereas the other is shown in surface mode. (C) Values for the accessible surface area (ASA), equivalent to solvent accessibility of each amino acid, are shown. Amino acid sites linked to mitochondrial localization are shown in red, whereas those associated with cytosolic localization are shown in green. With the exception of L10P, all of the DJ-1 mutations in the buried structure (ASA value <0.1) cause mitochondrial localization. (D,E) For quantitative analysis, the colocalization of DJ-1 mutants with the mitochondrial marker TOMM20 was calculated as a Pearson correlation coefficient in individual cells. (D) Cytosolic DJ-1 mutants; (E) mitochondrial mutants. Box plots are as described in the Materials and Methods section.

First, to identify the region important for cytosolic localization of DJ-1, we performed an alanine scan of the protein and examined the resulting subcellular localization in DJ-1-knockout HeLa cells (Kojima et al., 2016). Replacement of multiple sites (E16, V23, D24, R28, R48, C53, K63, N76, K89, K93, R98, T124, T125, H126, L128 and R156) had no effect on the cytosolic localization of DJ-1. In contrast, alanine replacement of E15, E18, R27, V33, T34, D68, or I105 resulted in mitochondrial translocation (Fig. 1A; Fig. S1A,B). The mutation sites of these new mitochondria-localized DJ-1 mutants (hereafter referred to as MLMs) are dispersed throughout the DJ-1 sequence, strongly suggesting that there is no ‘hotspot’ or restricted area in the DJ-1 structure that promotes mitochondrial localization. Using accessible surface area (ASA) view (Ahmad et al., 2004) to examine the correlation between the subcellular localization of each mutant and the solvent accessibility of the mutated amino acid position, we found that a majority of the MLM sites are buried in the DJ-1 structure (Fig. 1B,C), indicating that mitochondrial localization is correlated with DJ-1 instability. Consequently, we extended mutational analyses of the DJ-1 sequence to include various missense mutations that introduced amino acids with bulkier sidechains than alanine into the buried region. Although the initial alanine scan of some residues (E16A, V23A, T124A, and T125A) in the solvent inaccessible regions did not result in mitochondrial translocation (Fig. S1A), subsequent substitution with bulkier or less compatible amino acids (E16W, V23R, T124R, and T125R) generated the mitochondria-localized phenotype (Fig. 1A; Fig. S1A). Substitution with bulkier amino acids for A14, V44, A104, and C106 also caused mitochondrial localization (Fig. 1A). For quantitative colocalization and statistical analysis, the colocalization of various DJ-1 mutants with TOMM20 (a mitochondrial marker) in individual cells were calculated as a Pearson correlation coefficient. The Pearson correlation coefficients for 21 mutants classed as cytoplasmic were between 0 and 0.25 (Fig. 1D). Conversely, these values for the 22 mutants classed as mitochondrial were much higher and ranged from 0.6–0.9 (Fig. 1E). A number of mutants (I21T, S47A, H126A and P127A), however, had more varied values (0.1–0.6), suggesting they show variable or mixed localization (Fig. 1D; Fig. S1C). These quantitative analyses (Fig. 1D,E) are consistent with the immunocytochemical findings (Fig. 1A; Fig. S1). In total, ∼93% of mutations that were classed as buried in the DJ-1 structure (ASA value <0.1) caused mitochondrial localization, whereas ∼85% of the mutations that were classed as non-buried in the structure (ASA value >0.1) remained cytosolic (Fig. 1C). Therefore, we surmise that structural disturbances rather than disruption of specific regions of DJ-1 are the basis for the unique mitochondrial localization. Only one mutation (L10P) classed as buried in the DJ-1 structure (ASA value=0) remained cytosolic (Fig. 1C, arrowhead) and is studied in detail later in this paper.

### Stability of mitochondria-localized pathogenic DJ-1 mutants

**Fig. 2.**
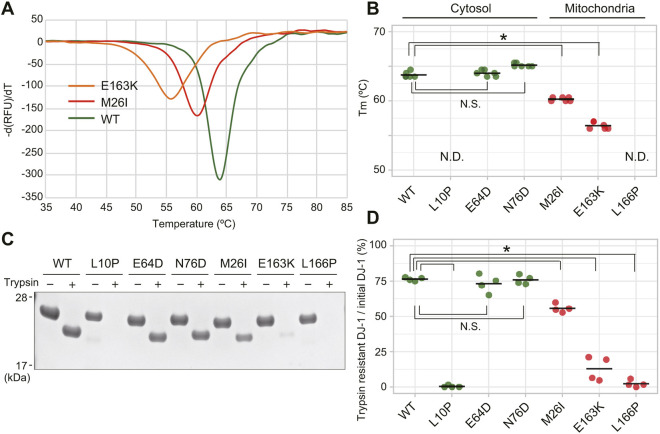
**Folding instability of DJ-1 pathogenic mutants determines their mitochondrial localization.** (A,B) The melting temperatures (Tm) of N-terminal 6×His-tagged DJ-1 proteins were measured via a thermal shift assay. Representative thermal spectra are shown in A, and averaged Tm with individual data points of three independent experiments are shown in B. Mitochondria-localized pathogenic DJ-1 mutants are characterized by a higher Tm. No Tm could be determined (N.D.) for the L10P mutation, suggesting the absence of any structure. (C,D) Folding stability of DJ-1 pathogenic mutants under physiological temperature (37°C) assessed via trypsin digestion. Recombinant 6×His-DJ-1 proteins with the indicated pathogenic mutations were incubated in the presence or absence of trypsin. (C) Representative immunoblotting data of two independent experiments are shown. (D) Degree of trypsin susceptibility after incubation for 22 h. Individual data points from two independent experiments are shown. *P<0.01, N.S., not significant (one-way ANOVA with Sidak's correction).

Next, we sought to investigate the stability of purified DJ-1 mutant proteins. We first focused on the pathogenic DJ-1 mutants. N-terminal 6xHis-tagged DJ-1 proteins with defined pathogenic mutations were purified and their folding stability was measured using thermal shift assays (Lo et al., 2004; Semisotnov et al., 1991). In this assay, recombinant DJ-1 is incubated with ThermoFluor, and the stability curve is obtained by gradually increasing the temperature and measuring the fluorescence. When DJ-1 unfolds, the exposed hydrophobic surfaces bind the ThermoFluor, resulting in an increase in fluorescence. Unfolding temperatures, which are analogous to melting temperatures (referred to as Tm), indicate the maximum value of the first derivative of the relative fluorescence unit (RFU) as a function of temperature (dRFU/dT) (Fig. 2A,B). Cytosolic pathogenic mutants had Tms that exceeded 63.5°C, whereas Tms for the mitochondrial localized mutants were significantly lower than the WT Tm and had a maximum Tm of 60.5°C. Two pathogenic mutants, L10P and L166P, had no detectable Tm, suggesting they are unable to initiate protein folding properly (Olzmann et al., 2004; Prahlad et al., 2014; Ramsey and Giasson, 2010). To further confirm destabilization of the DJ-1 mutants, we measured the sensitivity of each mutant to trypsin digestion under more physiological temperatures (normal body temperature 37°C). The pathogenic MLMs M26I, E163K and L166P were more susceptible to complete digestion than WT, suggesting that their structures are less ordered. Conversely, trypsin digestion of the cytosol-localized mutants E64D and N76D was comparable to WT (Fig. 2C,D). The thermal shift and trypsin digestion assays thus support differences between the folding states of cytosol-localized and mitochondria-localized pathogenic DJ-1 mutants. The exception to this conclusion, however, is the cytosolic mutant, L10P (Fig. 1D), which is highly sensitive to trypsin digestion (Fig. 2D) in addition to being thermally unstable (Fig. 2B). Although this mutant initially seems to conflict with our hypothesis that DJ-1 protein destabilization promotes mitochondrial translocation, additional analyses of this mutant (discussed in greater detail later) highlight the likelihood of a cryptic N-terminal mitochondria-targeting sequence.

Since DJ-1 is a causative gene for familial Parkinson's disease, we sought to assess whether the mislocalization of pathogenic DJ-1 mutants stresses mitochondria when expressed over long periods of time. To facilitate these analyses, we used OPA1, PGAM5 and ATF4 as mitochondrial stress indicators (Anand et al., 2014; Quiros et al., 2017; Sekine et al., 2012). When WT DJ-1 or mitochondria-localized pathogenic mutants E163K and L166P were expressed in DJ-1-knockout cells for 48 h, no difference was observed in the cleavage pattern of PGAM5 and OPA1, or on the induction of ATF4 (Fig. S2, lanes 1–4). Addition of the mitochondrial uncoupler valinomycin resulted in cleavage of both PGAM5 and OPA1 and induction of ATF4, indicating that the assay system was viable (Fig. S2, lanes 5 and 6). We thus conclude that the mitochondrial localization of the pathogenic DJ-1 mutants does not induce mitochondrial stress relevant to the onset of Parkinson's disease (discussed in greater detail later).

### Correlation between DJ-1 mutant instability and mitochondrial localization

**Fig. 3.**
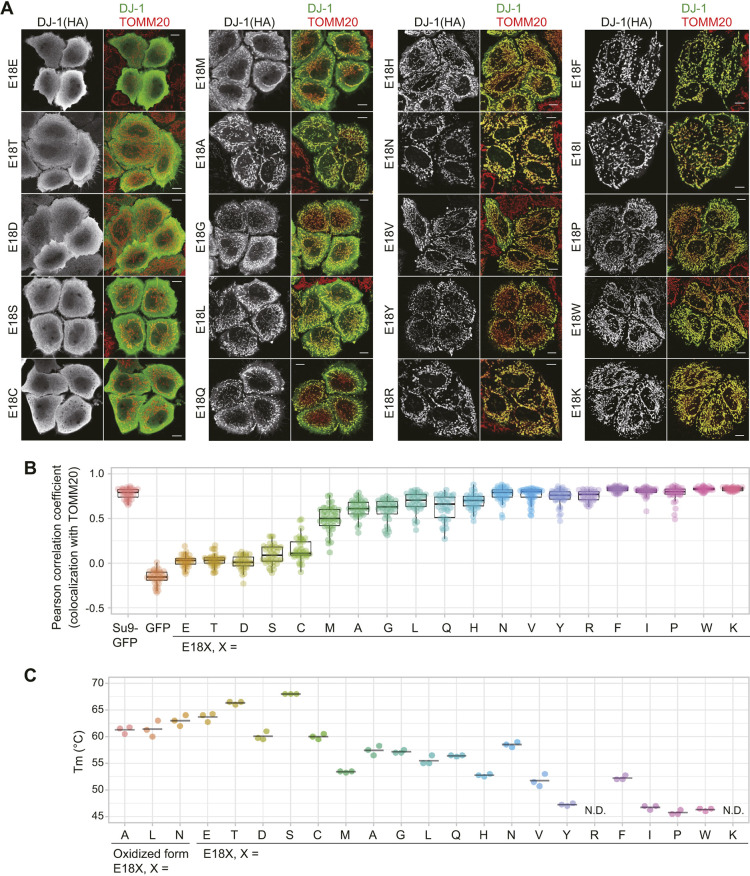
**Folding stability of DJ-1 E18X mutants.** (A) Systematic replacement of the E18 residue in DJ-1 leads to varied subcellular localization. Representative images of two independent experiments for the DJ-1 E18X (X indicates replacement amino acid) mutants are shown. The left figures show localization of the DJ-1 construct signal and the right figures show merged images for the DJ-1 constructs (green) and the mitochondrial marker TOMM20 (red) in DJ-1-knockout cells. Scale bars: 10 µm. (B) The Pearson correlation coefficients for colocalization of the DJ-1 E18X mutants with TOMM20 were calculated in individual cells. Su9–GFP and GFP are representative mitochondrial and cytosolic marker proteins. Individual data points of two independent experiments are shown. Box plots are as described in the Materials and Methods section. (C) Melting temperatures for the C-terminal 6×His-tagged E18X mutants were determined via thermal shift assays as in Fig. 2. Individual data points of three independent experiments and the averaged Tm for each construct are shown. N.D, not detected.

If our hypothesis that perturbations in protein folding affect DJ-1 localization is correct, then it should be possible to modulate the subcellular localization by altering the side-chain character of specific amino acids. To test this, we iteratively replaced E18, which resides within a central position in the DJ-1 structure, with other amino acids and assessed the effects on both protein stability and subcellular localization. Although localization of the E18D, E18N and E18Q mutants was previously reported as cytosolic (E18D) or mitochondrial (E18N and E18Q) (Blackinton et al., 2009), we generated all E18X mutants and assessed their localization when expressed in DJ-1-knockout HeLa cells. Overall, we found that the E18X mutants had three distinct localization patterns: exclusively cytosolic, exclusively mitochondrial, or a combination of both phenotypes (Fig. 3A). Because some mutants exhibited mixed localization, we tried to quantify the degree of localization for all E18X mutants. We calculated the Pearson correlation coefficient between the DJ-1 E18X mutants and TOMM20 for individual cells (Fig. 3B). The positive control, colocalization of Su9–GFP with TOMM20 [Su9 is also known as mitochondrial ATP synthase subunit 9 (atp-9) of *Neurospora crassa*], yielded a value of 0.8 (Fig. 3B, column 1), whereas the negative control, colocalization of cytosolic GFP with TOMM20, was ∼−0.2 (Fig. 3B, column 2). The coefficients for the mutants (E18T, E18D, E18S and E18C) that exhibited cytoplasmic localization comparable to WT were 0 to 0.3. Mutants with mixed cytoplasmic and mitochondrial localization (E18T, E18D, E18S and E18C) had coefficients of 0.5 to 0.7. In contrast, the values for the ten mutants (E18H, E18N, E18V, E18Y, E18R, E18F, E18I, E18P, E18W and E18K) that exclusively localized to mitochondria were 0.75 to 0.8, which is equivalent to the positive control (Su9–GFP). These data are consistent with the immunocytochemical findings (Fig. 3A,B). To examine the relationship between mitochondrial localization and DJ-1 destabilization, we purified recombinant proteins for all of the E18 mutants with a C-terminal 6×His-tag and examined their susceptibility to trypsin digestion. A clear correlation between trypsin sensitivity and mitochondrial localization was observed (Fig. S3A). For more precise quantification, we also measured the Tms of the E18X mutants (Fig. 3C; Fig. S3B). Although thermal spectra for most of the E18X mutants were characterized by a single peak (e.g. the E18T spectrum shown in Fig. S3B), those of the E18A, E18L and E18N mutants had two peaks, suggestive of bimodal unfolding transitions (Fig. S4). The Tms for these mutants were: E18A, 57.5°C and 61.2°C; E18L, 55.8°C and 61.8°C; and E18N, 58.5°C and 63°C. We speculate that the higher Tm transitions are due to oxidation of C106 given the reported presence of oxidized Cys106-SO_2_^−^ in the E18A and E18N DJ-1 mutants (Prahlad et al., 2014). The E18K and E18R mutants had no detectable unfolding transition, suggesting they are largely unstructured. We found that the E18X mutants that localize to the cytosol (E18S, E18T, E18C and E18D) had Tms of more than 60°C, whereas a majority of the exclusively mitochondria-localized mutants (E18H, E18F, E18Y, E18I, E18W, E18P, E18K and E18R) had substantially reduced thermal stabilities (>11°C) compared to WT (Fig. 3C). These data clearly indicate that DJ-1 instability directly correlates with the mitochondrial localization state.

### Destabilized DJ-1 mutants localize inside the mitochondrial matrix

**Fig. 4.**
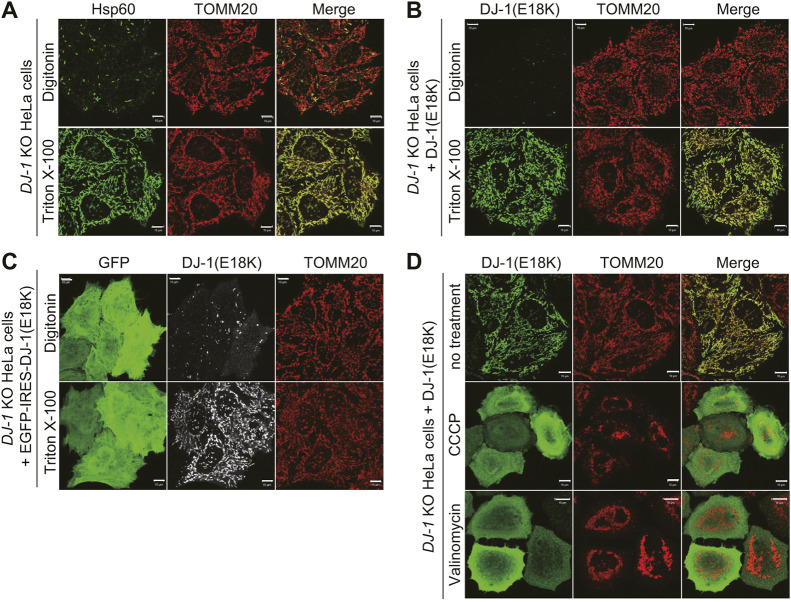
**The DJ-1 E18K mutant is translocated into the mitochondrial matrix.** (A) Permeabilization with digitonin was insufficient for detecting the matrix protein Hsp60, instead permeabilization with Triton X-100 was required. In contrast, the outer mitochondrial membrane protein TOMM20 was observed irrespective of the permeabilization conditions. (B) DJ-1-knockout (KO) HeLa cells expressing an HA-tagged DJ-1 E18K mutant were immunostained with an anti-HA antibody after permeabilization with digitonin or Triton X-100. Detection of the DJ-1 E18K mutant requires Triton X-100 permeabilization. (C) To confirm that the DJ-1 E18K mutant was expressed in digitonin-permeabilized cells, a GFP–IRES–DJ-1(E18K) plasmid was transfected and subjected to immunocytochemistry as in B. (D) DJ-1 KO HeLa cells were pre-treated with 10 µM valinomycin or 15 µM CCCP prior to transfecting HA-tagged DJ-1 E18K. The subcellular localization was determined using anti-HA and anti-TOMM20 antibodies. Valinomycin and CCCP treatment inhibited mitochondrial localization of the DJ-1 E18K mutant. Data shown here indicate that DJ-1 is transported across the inner mitochondrial membrane. Reproducibility of these results were confirmed by two independent experiments. Scale bars: 10 µm.

The dataset presented so far does not rule out the trivial possibility that the DJ-1 mutants are attached to the mitochondrial surface rather than being translocated to the mitochondrial matrix. We thus sought to confirm matrix localization of the HA-tagged DJ-1 mutants. First, we assessed the utility of different permeabilization methods for immunocytochemical analysis. Triton X-100 (1%) permeabilizes both the outer mitochondrial membrane (OMM) and inner mitochondrial membrane (IMM) and enables antibodies to access matrix proteins such as mitochondrial Hsp60 (also known as HSPD1), whereas digitonin (50 µg/ml) does not sufficiently permeabilize the IMM (Kojima et al., 2016; Okatsu et al., 2015a) for Hsp60 detection (Fig. 4A). For the E18K mutant, anti-HA immunoreactivity was not observed in the mitochondria after permeabilization with digitonin but was detectable following Triton X-100 permeabilization (Fig. 4B). Since the DJ-1 E18K mutant was exogenously transfected into cells, it is possible that the DJ-1(E18K) was not fully introduced in the digitonin-permeabilized cells. To address this concern, cells were transfected with a GFP–IRES–DJ-1(E18K) plasmid and then subjected to immunocytochemistry. GFP–IRES–DJ-1(E18K) guarantees the expression of the E18K mutant in GFP-expressing cells. In the GFP-positive cells, the E18K mutant-derived signal remained undetectable following digitonin permeabilization but, as before, was present with Triton X-100 permeabilization (Fig. 4C). These results confirmed mitochondrial matrix localization of the mutant.

The dependence of DJ-1 translocation on the mitochondrial membrane potential (ΔΨm) also supports mitochondrial matrix localization. The ΔΨm is generated across the IMM and is essential for canonical matrix-localized proteins to pass the IMM (Hartl et al., 1989). Treatment of cells with uncouplers such as valinomycin and carbonyl cyanide *m*-chlorophenyl hydrazine (CCCP) dissipate the ΔΨm. Pre-treating cells with either of the two uncouplers prior to transfecting the DJ-1 E18K mutant resulted in cytosolic localization rather than the mitochondrial localization typical of the mutant (Fig. 4D). These results indicate that the DJ-1 E18K mutant is imported into the matrix in a ΔΨm-dependent manner.

### Mitochondrial translocases facilitate localization of DJ-1 mutants to the IMM

**Fig. 5.**
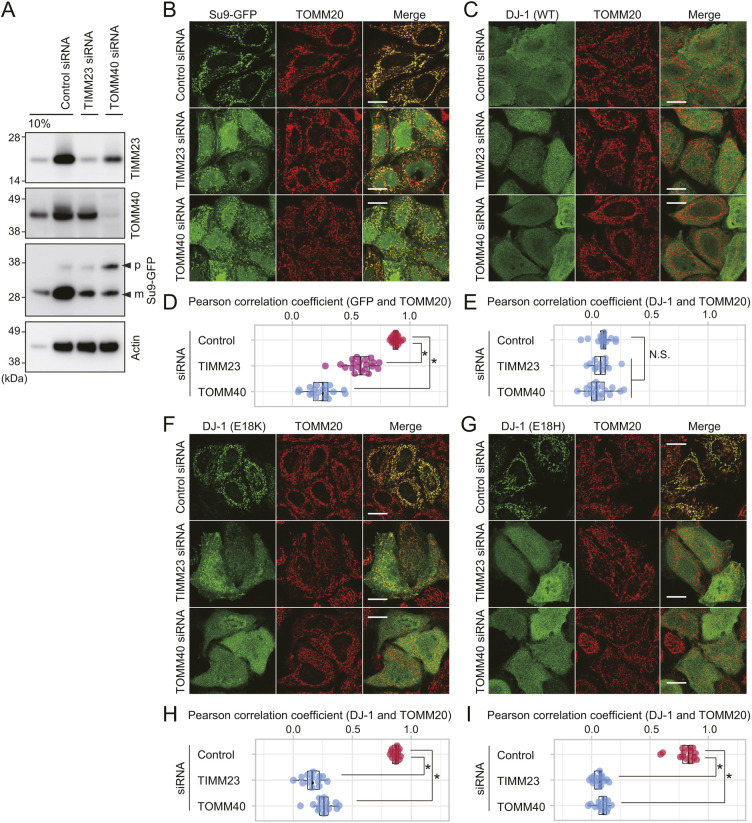
**Import of the DJ-1 mutants requires mitochondrial protein translocases TOMM40 and TIMM23.** (A) siRNA-mediated reduction in expression of endogenous TIMM23 and TOMM40. DJ-1 KO HeLa cells expressing Su9–GFP were treated with control, TIMM23, or TOMM40 siRNAs, and then immunoblotted using the indicated antibodies. To assess the knockdown efficiency, 10% of the control siRNA treated cell lysate was also loaded. The precursor and mature forms of Su9–GFP are shown as p and m. Immunoblots are representative of two independent experiments. (B,C) DJ-1 KO HeLa cells pre-treated with control, TIMM23 or TOMM40 siRNA were transfected with Su9–GFP (B) or WT DJ-1 (C), and then subjected to immunocytochemistry. (D,E) For quantitative analysis of the colocalization of GFP or DJ-1 with TOMM20, the Pearson correlation coefficient in individual cells was calculated, with quantitative analyses done using data in B and C. (F–I) DJ-1 KO HeLa cells pre-treated with control, TIMM23, or TOMM40 siRNA were transfected with E18K (F,H) or E18H (G,I) DJ-1 mutants and then subjected to immunocytochemical and quantitative analyses as in B–E. *P<0.01, N.S., not significant compared to control (one-way ANOVA with Sidak's correction). Box plots are as described in the Materials and Methods section. Representative images of two independent experiments are shown in B, C, F and G. Scale bars: 10 µm.

Mitochondrial matrix protein precursors are imported into the matrix by sequential passage through TOMM40 (a core channel component of translocases on the outer membrane complex; the TOMM complex) and TIMM23 (a core channel component of the inner membrane complex; the TIMM23 complex). Since TOMM40 and TIMM23 are essential genes, functional analysis of their role in DJ-1 E18K mitochondrial localization could not be performed using knockout cell lines. Instead, we utilized small interfering RNA (siRNA)-mediated knockdown to suppress expression of the endogenous TOMM40 and TIMM23 proteins (Fig. 5A). As a model precursor protein for targeted matrix import, we used Su9–GFP, which utilizes the N-terminal region of F_o_ ATPase subunit 9 (Su9) to direct GFP to the matrix via the TOMM and TIMM23 complexes (Ishihara et al., 2006). Knockdown of either TOMM40 or TIMM23 by siRNA reduced the mitochondrial localization of Su9–GFP (Fig. 5B). Quantification of the degree of colocalization between Su9–GFP and TOMM20 confirmed that the mitochondrial translocation of Su9–GFP was reduced following knockdown of TOMM40 and TIMM23 (Fig. 5D). A decrease in the mature (processed) form of Su9–GFP was also observed (Fig. 5A). These results confirmed that knockdown of TOMM40 and TIMM23 impedes protein import into the matrix. For WT DJ-1, cytosolic localization was not affected in either the immunocytochemical or quantification-based analyses following knockdown of TOMM40 or TIMM23 (Fig. 5C,E). Conversely, mitochondrial localization of the E18K mutant was clearly inhibited following knockdown of TOMM40 or TIMM23 in the immunocytochemical images (Fig. 5F), and these results were further substantiated by quantitative analysis (Fig. 5H). Mitochondrial localization of the E18H mutant was likewise inhibited (Fig. 5G,I). The immunocytochemical data shown in Figs 4 and 5, in conjunction with previous reports describing matrix localization of DJ-1 E18A/H126A mutants (Kojima et al., 2016; Maita et al., 2013), indicate that matrix localization of DJ-1 mutants depends on TOMM40, TIMM23 and an intact ΔΨm.

### The N-terminus of DJ-1 functions as a destabilization-dependent MTS

**Fig. 6.**
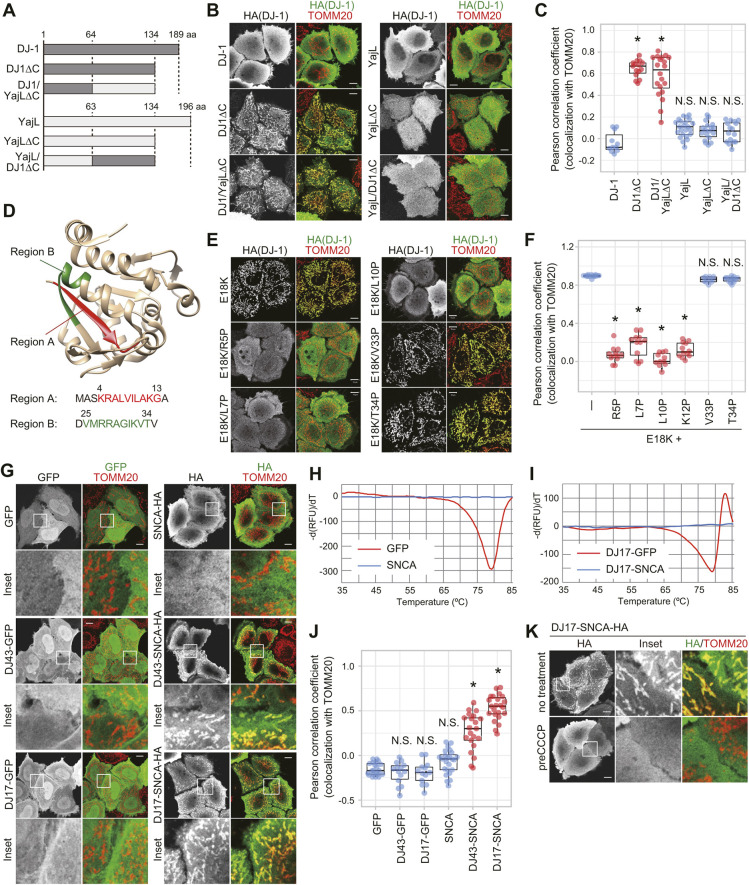
**DJ-1 has a cryptic N-terminal MTS that is essential for mitochondrial localization.** (A) Schematic diagram of the DJ-1 and YajL chimeric constructs. (B) Subcellular localization of the chimeric proteins in DJ-1 knockout cells. (C) Quantitative analysis of data shown in B. The colocalization of various chimeric proteins with the mitochondrial marker TOMM20 was calculated as a Pearson correlation coefficient in individual cells. (D) Sequence of regions A (red) and B (green) and their location in the DJ-1 structure. Those regions were detected by determining the PA score used in MitoFates. (E) Proline mutations in region A of the E18K mutant inhibit mitochondrial localization in DJ-1-knockout cells, whereas the same mutations in region B had no effect on mitochondrial localization. (F) Quantitative analysis of data shown in E. The colocalization of DJ-1 mutants with TOMM20 was calculated as a Pearson correlation coefficient in individual cells. (G) The subcellular localization of GFP and α-synuclein (SNCA) when fused with amino acids 1–43 (DJ43) or 1–17 (DJ17) of DJ-1. SNCA is targeted to the mitochondria when fused with the DJ-1 MTS, whereas the same MTS had no effect on GFP localization. (H,I) Thermal spectra of GFP and SNCA (H) or DJ17–GFP and DJ17–SNCA (I). Negative peaks in the thermal spectra indicate high melting temperatures (Tm) for GFP and DJ17–GFP, whereas the absence of peaks in SNCA and DJ17–SNCA suggests they are intrinsically disordered proteins. (J) Quantitative analysis of G. The colocalization of chimeric proteins with TOMM20 was calculated as a Pearson correlation coefficient in individual cells. (K) Uncoupler treatment inhibited the mitochondrial localization of DJ17–SNCA. In B, E, G and K, representative images of two independent experiments are shown. The left images show localization of the specific chimeric or mutant proteins alone, whereas images to the right show merged images for the chimeric or mutant proteins (green) and TOMM20 (red). *P<0.01, N.S., not significant compared to control (one-way ANOVA with Sidak's correction). Box plots are as described in the Materials and Methods section. Scale bars: 10 µm.

We next tried to identify the region responsible for mitochondrial localization of the DJ-1 MLMs. To narrow down the DJ-1 mitochondrial localization domain, we performed domain-swaps with a prokaryotic homolog of human DJ-1, the *Escherichia coli* protein YajL (Fig. 6A). YajL and DJ-1 have 38% amino acid sequence identity and their crystal structures have almost identical backbone structures with a Cα root-mean-square deviation (RMSD) value of two angstroms (1 angstrom=0.1 nm) (Wilson et al., 2005). When expressed in DJ-1-knockout HeLa cells, both WT DJ-1 and YajL are cytosolic (Fig. 6B, top panels). C-terminal deletion of DJ-1 (removal of residues 135–189, abbreviated as DJ-1ΔC) promoted mitochondrial translocation, whereas deletion of the same region of YajL (removal of residues 135–196, abbreviated as YajLΔC) did not affect its cytosolic localization (Fig. 6B, middle panels). We thus assessed the effects of domain-swaps between YajLΔC (cytosolic localization) and DJ-1ΔC (mitochondrial localization) to identify the essential mitochondrial localization region of DJ-1ΔC. An N-terminal DJ-1 and C-terminal YajL chimera (abbreviated as DJ1/YajLΔC) localized to the mitochondria, whereas the reciprocal chimera, N-terminal YajL and C-terminal DJ-1 (abbreviated as YajL/DJ1ΔC), was cytosolic (Fig. 6B, bottom panels). Pearson-based quantitative analysis confirmed the immunocytochemical findings (Fig. 6C). Thus the DJ-1 N-terminal region (residues 1–64), which was sufficient to drive translocation of YajL(64-134) to the mitochondria, functions as a novel type of signal for mitochondrial translocation.

Based on previous structural data (Honbou et al., 2003; Olzmann et al., 2004; Tao and Tong, 2003; Wilson et al., 2003), it is clear that DJ-1 does not possess typical MTS structures that form an amphiphilic α-helical structure with hydrophobic residues on one side and positively charged residues on the opposite side. However, when the positively charged amphiphilicity score (PA score) from the novel prediction algorithm MitoFates was considered in our search for a cryptic mitochondria-targeting signal (Fukasawa et al., 2015), two candidate regions corresponding to residues 4–13 (region A) and residues 25–34 (region B) were predicted (Fig. 6D). To determine whether these regions are responsible for the mitochondrial localization of the DJ-1 mutants, we introduced mutations into both regions of DJ-1(E18K) and examined whether the mutation affected mitochondrial localization. We showed above that single mutations (R27A, V33A or T34A) in region B (Fig. 1A) in the absence of the E18K mutation promoted mitochondrial localization of DJ-1, suggesting that the WT residues do not comprise the mitochondrial targeting motif. Furthermore, when V33 and T34 were replaced with proline, no change in the mitochondrial localization of the E18K mutant was observed (Fig. 6E, right column). In contrast, when R5, L7, L10 or K12 in region A were replaced with proline, mitochondrial localization of the E18K mutant was clearly impeded (Fig. 6E,F), indicating that region A is responsible for mitochondrial targeting of MLMs. This finding also explains the initial discrepancy between protein stability and cytosolic localization of the L10P mutation shown in Figs 1 and 2, as L10 comprises a portion of the cryptic signal in Region A that is essential for mitochondrial import.

In general, to prove that a sequence is a MTS, we need to show that addition of the sequence to a cytoplasmic marker protein (such as GFP) redirects localization to the mitochondria. For example, the N-terminal region of Su9 targets GFP to the mitochondria (Fig. 5A,B). To confirm that the N-terminal region of DJ-1 functions as a MTS, we fused amino acids 1–43 of DJ-1 to GFP and examined its subcellular localization. The fusion (DJ43–GFP), however, did not change the cytosolic localization of GFP (Fig. 6G). This result is consistent with our previous data showing that fusion of GFP to the C-terminus of DJ-1 mutants (E18A, M26I and L166P) did not promote mitochondrial localization, even though both HA-tagged and non-tagged DJ-1 mutants harboring the same mutations localized to mitochondria (Kojima et al., 2016). Given its destabilization dependency, we speculated that the nature of the DJ-1 MTS differs from the typical MTS. We thus switched to a less stable marker protein, α-synuclein (SNCA), which unlike the stable GFP structure (Ormo et al., 1996), is naturally unstructured and does not form a specific conformation (Theillet et al., 2016). Indeed, recombinant GFP had a high Tm value (Tm=79°C) in our thermal shift analysis, while the Tm for recombinant SNCA could not be defined (Fig. 6H). Under steady-state conditions, SNCA is cytosolic but the addition of DJ-1 residues 1–43 promoted mitochondrial import (Fig. 6G, DJ43–SNCA). Moreover, fusion with a smaller segment of the DJ-1 N-terminus that was largely limited to region A (residues 1–17) was sufficient for mitochondrial localization of SNCA (Fig. 6G, DJ17–SNCA), whereas the same region had no effect on GFP localization (Fig. 6G, DJ17–GFP). As expected, the thermal shift analysis confirmed that DJ17–GFP was tightly folded (Tm=79°C), whereas DJ17–SNCA was unfolded with an undetectable Tm (Fig. 6I). Pearson-based quantitative analysis confirmed that region A of DJ-1 has stronger mitochondria-localization activity than the larger 43-amino-acid sequence (Fig. 6J). Mitochondrial localization of DJ17–SNCA was completely inhibited by CCCP treatment (Fig. 6K), indicating that the DJ-1 N-terminus does not attach SNCA to the surface of mitochondria, but rather transports the protein into the mitochondrial matrix. Taken together, these results demonstrate a functional role for region A as a conformation-dependent MTS.

### Dual degradation systems for destabilized DJ-1

**Fig. 7.**
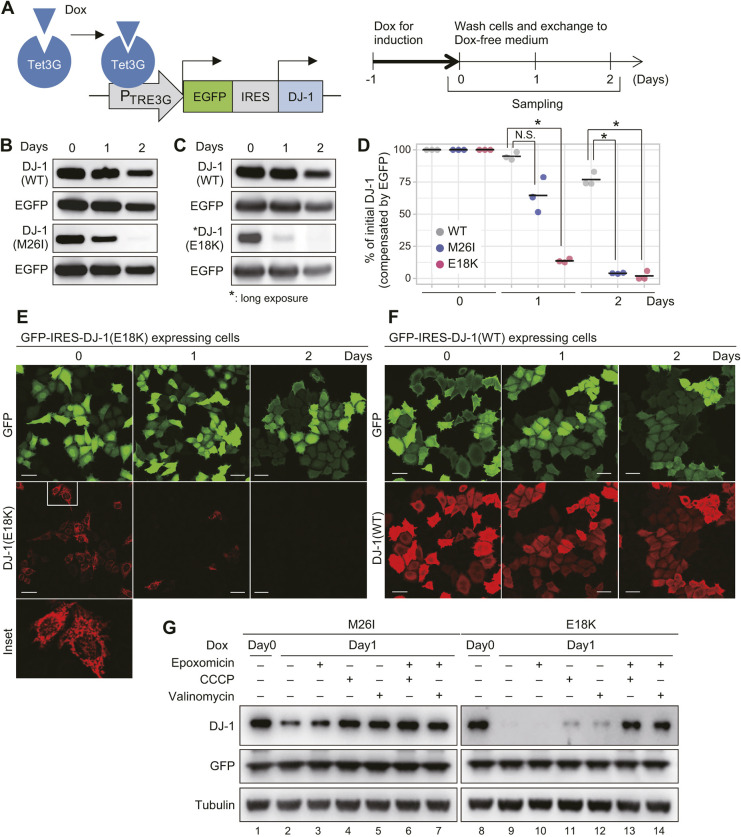
**Mitochondrial import leads to DJ-1 degradation in mitochondria.** (A) Scheme for the Dox inducible system. (B,C) Changes in the amount of the DJ-1 mutant proteins in DJ-1 knockout HeLa cells. Immunoblotting data for M26I (B) and E18K (C) with WT and EGFP controls are shown. Data are representative of three independent experiments. (D) Degree of protein degradation for the WT, M26I, and E18K mutants in relation to the internal EGFP control. Individual data points of three independent experiments are shown. *P<0.01, N.S., not significant (one-way ANOVA with Sidak's correction) compared to WT. (E,F) Images depicting immunocytochemistry profile of cells expressing GFP–IRES–DJ-1. When E18K was co-expressed with GFP, cells positive for both GFP and mitochondrial E18K rapidly disappeared within 1 day (E). In contrast, the double-positive cells persisted when WT DJ-1 was co-expressed with GFP (F). Reproducibility of data in E and F were confirmed in three independent experiments. Scale bars: 40 µm. (G) Inhibition of both mitochondrial import and proteasome activity is essential for stabilization of the DJ-1 E18K mutant. Immunoblots are representative of two independent experiments.

To investigate the fate of mitochondria-localized denatured DJ-1 proteins, we established an experimental system in which an internal ribosome entry site (IRES)-connected DJ-1 and GFP (an internal control) were simultaneously and transiently expressed following doxycycline (Dox) induction (Fig. 7A). Using this system, transient expression of DJ-1 mutants was induced for 24 h with Dox treatment in DJ-1-knockout HeLa cells, which was then removed, and differences in the amount of DJ-1 protein were assessed. WT DJ-1 was stable and clearly observed 48 h post Dox induction (Fig. 7B,C, upper two panels). In contrast, the E18K and M26I mutants, which undergo mitochondrial translocation, were not observed at the later time point (Fig. 7B,C, lower two panels). After normalizing to the GFP internal control, protein quantification revealed that the level of WT DJ-1 underwent a modest 5% reduction at 24 h post-induction, whereas that of the M26I mutant decreased 35% and the E18K mutant ∼87% (Fig. 7D). Immunocytochemical analysis confirmed these results. In cells co-expressing E18K and GFP, a significant percentage (∼45–55%) of cells were initially positive for both GFP and mitochondrial DJ-1; however, most of the mitochondrial signal disappeared 24 h post Dox induction (Fig. 7E). In contrast, in cells co-expressing WT DJ-1 and GFP, both DJ-1 and GFP were clearly observed 48 h post Dox induction (Fig. 7F). These results suggest that destabilized DJ-1 proteins are degraded after mitochondrial translocation. Consistent with this, a recent report found that a number of pathogenic DJ-1 mutants localized in mitochondria undergo intra-mitochondria degradation mediated by LonP1 (Sanchez-Lanzas and Castano, 2021).

We next examined whether this degradation is reduced when mitochondrial import is blocked. After Dox-mediated induction, cells were treated with CCCP or valinomycin to inhibit mitochondrial translocation (Fig. 4D). Although the M26I mutant accumulated following CCCP and valinomycin treatment (Fig. 7G, compare lane 2 with lanes 4 and 5), the E18K mutant was barely detectable (Fig. 7G, compare lane 9 with lanes 11 and 12). When the two mutants are compared, the E18K mutation generates a protein that is less stable both *in vitro* (Fig. 3; Fig. S3) and in cells (Fig. 7B,C), and which undergoes more complete mitochondrial localization (Figs 1 and 3). The results shown in Fig. 7G thus seem contradictory. A clue to understanding this discrepancy was provided by the L10P mutant, which, although denatured (Fig. 2), cannot undergo mitochondrial import because of a mutated MTS (Fig. 6). The E18K mutant is similarly denatured but its mitochondrial import is only inhibited following CCCP or valinomycin treatment. Because the L10P mutant undergoes proteasomal degradation (Ramsey and Giasson, 2010; Sanchez-Lanzas and Castano, 2021), we examined the effects of the proteasome inhibitor epoxomicin on mutant DJ-1 protein levels in cells treated with CCCP and valinomycin. Interestingly, epoxomicin failed to inhibit degradation of the E18K mutant (Fig. 7G, lane 10), and only a slight increase in the levels of the mutant protein was observed following CCCP or valinomycin alone (Fig. 7G, lanes 11 and 12). In contrast, a dramatic increase in E18K protein levels was achieved by simultaneous treatment with either uncoupler (CCCP or valinomycin) and the proteasome inhibitor (Fig. 7G, lanes 13 and 14), indicating that denatured E18K is usually degraded intra-mitochondrially but undergoes proteasomal degradation if cytosolic.

### The mitochondrial localization of DJ-1 mutants is reversed by a DJ-1 stabilizer

**Fig. 8.**
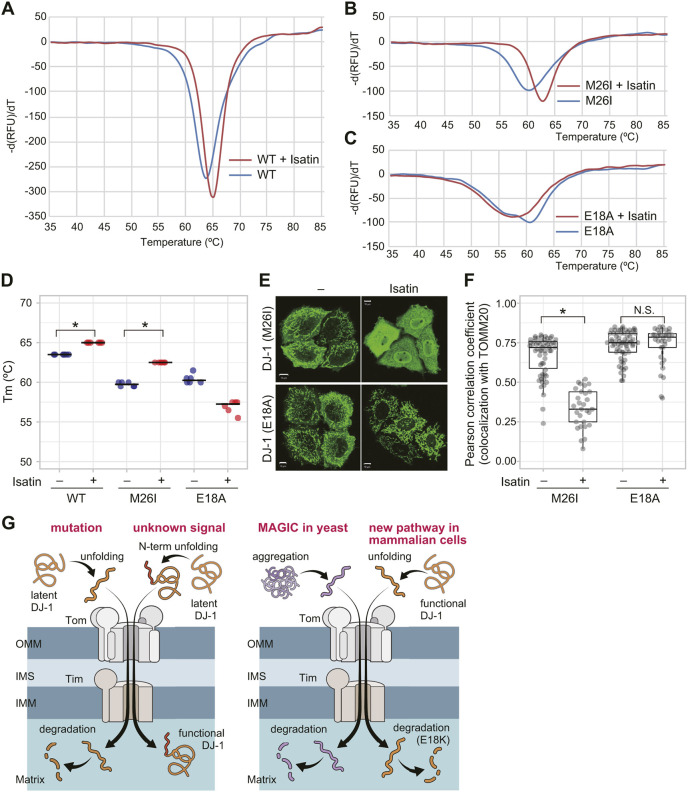
**DJ-1 stabilization reverses mitochondrial localization of DJ-1 mutants.** (A–C) Representative thermal shift spectra for WT DJ-1 (A), M26I (B), or E18A (C) mutant in the presence of isatin. Isatin stabilizes and increases the melting temperature (Tm) of the M26I mutant but does not stabilize the E18A mutant. (D) Quantified data of the thermal shift assay are shown. Individual data points of three independent experiments are shown. *P<0.01 compared to untreated control (one-way ANOVA with Sidak's correction). (E) The subcellular localization of E18A and M26I mutants in the absence or presence of isatin when expressed in DJ-1-knockout HeLa cells. Representative images of two independent experiments are shown. Scale bars: 10 µm. (F) The colocalization of DJ-1 M26I or E18A mutants with TOMM20 following isatin treatment was calculated as a Pearson correlation coefficient in individual cells. *P<0.01, N.S., not significant 01 compared to untreated control (one-way ANOVA with Sidak's correction). Isatin stabilization of the DJ-1 M26I mutant restores its cytoplasmic localization, whereas the isatin-insensitive E18A mutant remains localized in mitochondria. Box plots are as described in the Materials and Methods section. (G) Two possible schematic models for DJ-1 translocation into mitochondria. Left panel; restricted unfolding by unknown signal and subsequent mitochondrial translocation is essential for the genuine function of DJ-1, and that the mitochondria-localized DJ-1 mutants reflect this phenomenon. Right panel; mitochondrial localization of DJ-1 reflects an undetermined mitochondria-based quality control system for cytoplasmic proteins like “MAGIC” in yeast cells. See text for details.

If protein unfolding is the driving force for the mitochondrial localization of DJ-1, then artificial stabilization should impact DJ-1 localization. We thus examined whether mitochondrial localization of DJ-1 mutants could be blocked with a DJ-1 stabilizer. Using a thermal shift assay, we determined that isatin [a small-molecule DJ-1 inhibitor that covalently binds to the DJ-1 C106 catalytic center (Tashiro et al., 2018)] stabilizes both WT DJ-1 and the M26I mutant. Isatin interactions caused a slight increase (ΔTm=1.5°C) in the WT Tm (Fig. 8A,D) and a significant increase (ΔTm=2.5°C) in Tm for the M26I mutant (Fig. 8B,D). When DJ-1-knockout HeLa cells were treated with isatin, the mitochondrial localization of M26I was significantly inhibited (Fig. 8E,F). These results, however, do not rule out the possibility that isatin affects M26I translocation by decreasing ΔΨm similar to CCCP, or that it inhibits some factor that functions in DJ-1 translocation such as the TOMM or TIMM complex. Serendipitously, we found that rather than stabilizing the E18A mutant, isatin actually destabilizes E18A (Fig. 8C,D). As such, unlike the M26I mutant, the mitochondrial localization of E18A was unaffected by isatin in DJ-1-knockout HeLa cells (Fig. 8E,F). These results indicate that the effects of the compound on the M26I mutant result from protein stabilization rather than via inhibition of a process in mitochondrial translocation, thus supporting our hypothesis that protein unfolding is the driving force underlying DJ-1 mitochondrial localization.

## DISCUSSION

A close relationship between neurodegenerative disease and impaired mitochondrial function has been widely recognized. Parkinson's disease is the best-elucidated example with diverse familial Parkinson's disease-associated genes implicated in mitochondrial quality control. One of those genes, *PARK7*, encodes the cytosolic protein DJ-1. Some pathogenic mutations unexpectedly trigger DJ-1 mislocalization from the cytosol to the mitochondrial matrix. Translocation of steady-state cytosolic proteins to the mitochondrial matrix by missense mutations is rare and the underlying mechanism that causes the mitochondrial import has yet to be elucidated.

Mitochondrial translocation of DJ-1 is triggered by several pathogenic mutations, such as L166P (Bonifati et al., 2003; Kojima et al., 2016; Maita et al., 2013; Ren et al., 2012). Because other causal gene products for recessive familial Parkinson's disease, such as PINK1 and Parkin, localize on damaged mitochondria, one might assume that the mechanisms driving the mitochondrial localization of DJ-1, PINK1 and Parkin are similar. However, as a critical point, we want to stress that mitochondrial translocation is not essential for DJ-1 mutants to trigger the onset of Parkinson's disease. In the case of PINK1 and Parkin, their enzymatic activities as a ubiquitin kinase and ubiquitin ligase manifest only on damaged mitochondria (Kane et al., 2014; Kazlauskaite et al., 2014; Koyano et al., 2014; Matsuda et al., 2010; Narendra et al., 2008, 2010; Okatsu et al., 2015b; Yamano et al., 2016). Indeed, mitochondrial localization is essential for PINK1 and Parkin to exert their genuine functions, inhibition of which promotes Parkinson's disease. Consequently, pathogenic mutations impede their mitochondrial localization. In contrast, various pathogenic mutations in DJ-1 accelerate rather than inhibit mitochondrial localization. The absence of observable mitochondrial stress induced by mitochondria-localized pathogenic DJ-1 mutants (Fig. S2), however, suggests that they are likely not directly relevant to the onset of Parkinson's disease. Rather, this study demonstrates that destabilized DJ-1 mutants, including several pathogenic mutations (M26I, E163K and L166P), translocate to mitochondria, and that a new mitochondrial transport mechanism, which we term ‘destabilization-dependent mitochondrial import mechanism’, drives their mitochondrial localization.

We reported here that the destabilizing effects of various mutations on the DJ-1 protein conformation trigger its translocation to mitochondria. To our knowledge, there are only a few reports of unfolding-dependent mitochondrial translocation. In terms of Parkinson's disease, it is unlikely that pathogenic mitochondrial DJ-1 mutants (such as M26I, E163K or L166P) exert an effect after transport to the mitochondria. Since these DJ-1 mutants are translocated to mitochondria in response to unfolding, they are likely non-functional and subsequently destined for degradation (Fig. 7). However, if mitochondrial import is only a process in DJ-1 dysfunction, then the question of why DJ-1 has the potential/capacity to undergo mitochondrial translocation remains. As suggested by the fact that mitochondrial translocation of DJ-1 is caused by recessive pathogenic mutations (i.e. loss-of-function mutations), it could be that mitochondrial transport is simply a phenomenon accompanying DJ-1 dysfunction. Conversely, mitochondrial transport might have a positive and significant role on DJ-1. Regarding this topic, we can consider two possibilities. The first possibility is that restricted unfolding and subsequent mitochondrial translocation is essential for the genuine function of DJ-1, and that the DJ-1 MLMs reflect this phenomenon (Fig. 8G, left). As discussed above, the cryptic mitochondrial localization signal at the N-terminus of DJ-1 is essential for the protein to translocate to mitochondria following unfolding. It is possible that some unknown signal or ligand affects the DJ-1 structure restrictedly, and the resulting structural change allows the cryptic N-terminal signal to be exposed. Interestingly, Prahlad et al. reported that E18A, E18N and E18Q, which localize in mitochondria (Fig. 3), are fully dimeric, suggesting that they are well-folded, but are also more structurally dynamic and flexible than WT DJ-1 (Prahlad et al., 2014). That could be consistent with our model, where structural fluctuations in more dynamic mutants transiently expose the N-terminal region and permit mitochondrial import.

A second, and completely different, possibility is that mitochondrial localization of DJ-1 reflects an undetermined mitochondria-based quality control system for cytoplasmic proteins. Interestingly, a similar unfolding-dependent mitochondrial translocation and degradation mechanism, dubbed ‘mitochondria as guardian in cytosol’ (MAGIC), has been reported in yeast (Ruan et al., 2017). In the yeast model, aggregation-prone proteins become disentangled and flood into the mitochondrial matrix. The authors of that study reported on the presence of a similar phenomenon in human retinal pigment epithelium 1 (RPE-1) cells in which a super unstable variant of Flag-tagged luciferase was funneled into the mitochondrial matrix. Data in support of MAGIC in mammalian cells, however, has yet to be further developed. Here, we reported DJ-1 transport into mitochondria via a MAGIC-like mechanism in mammalian cells. As indicated, the reported substrate of mammalian MAGIC was an artificial model protein, and thus DJ-1 could be the first endogenous substrate protein for this mechanism. We believe mutant DJ-1 provides a reproducible and reliable experimental model for exploring MAGIC (which is a novel but not well-accepted idea) in mammalian cells (Fig. 8G, right). This destabilization-dependent import may be the reason why aggregation-prone proteins, such as Aβ and TDP43, have also been reported in mitochondria despite the absence of clear mitochondrial localization signals (Devi et al., 2006; Izumikawa et al., 2017; Manczak et al., 2006; Wang et al., 2016).

## MATERIALS AND METHODS

### Cell culture and transfection

HeLa cells were cultured at 37°C with 5% CO_2_ in Dulbecco's modified Eagle's medium (DMEM, Sigma-Aldrich) containing 1× nonessential amino acids (Gibco), 1× sodium pyruvate (Gibco) and 10% fetal bovine serum (Gibco). HeLa cells used in this study (Cell No. KBN0573-01) were authenticated by the Japanese Collection of Research Bioresources Cell Bank (JCRB) cell bank at the National Institute of Biomedical Innovation (Osaka, Japan) as being the same as the HeLa cell registered in ATCC. DJ-1-knockout cells were produced using CRISPR as previously described (Kojima et al., 2016). Transfection of plasmids for transient expression was undertaken using the FuGENE6 transfection reagent (Promega) according to the manufacturer's protocol. Plasmids used are given in Table S2.

### Immunocytochemistry

Cells were fixed with 4% paraformaldehyde, permeabilized in 1% Triton X-100 or 50 µg/ml digitonin for 15 min, diluted in blocking solution (0.1% gelatin in PBS), blocked for 30 min, and stained with the following primary antibodies: an anti-TOMM20 antibody FL-145 (Santa Cruz Biotechnology, 1:2000), an anti-Hsp60 antibody N20 (Santa Cruz Biotechnology, 1:250), or an anti-HA antibody TANA2 (MBL, 1:1000). Among the secondary antibodies used were an Alexa Fluor 488-, 568- or 647-conjugated anti-mouse IgG or an anti-rabbit IgG antibody (Thermo Fisher Scientific, 1:2000). Cells were imaged using a laser-scanning microscope (LSM710 or LSM780; Carl Zeiss). Antibodies used in this study and their Research Resource Identifiers (RRIDs) are listed in Table S1.

### Uncoupler treatment to depolarize mitochondria

To examine whether the mitochondrial transport of the DJ-1 E18K mutant and DJ17–SNCA depends on ΔΨm, HeLa cells were pre-treated with 10 µM CCCP (Sigma) or 10 µM valinomycin (Sigma) for 2 h before transfection, and then the plasmid encoding the DJ-1 E18K mutant or DJ17–SNCA was introduced. Cells were further incubated for 20 h in the presence of CCCP or valinomycin, and the subcellular localization of DJ-1 E18K or DJ17–SNCA was examined.

### siRNA analyses

For siRNA analysis, the siGENOME siRNA SMART pool TIMM23 (M-190121-00, Thermo Fisher Scientific) and siGENOME siRNA SMART pool TOMM40 (M-012732-00, Thermo Fisher Scientific) were used for knockdown of TIMM23 and TOMM40, respectively. As a control, the siGENOME Control siRNA pool (D-001206-13-20, Thermo Fisher Scientific) was used. 10 nM TIMM23, TOMM40 and control siRNAs were introduced into HeLa cells using Lipofectamine RNAiMAX (Life Technologies). At 24 h post siRNA transfection, the cells were re-seeded on 35 mm glass bottom dishes (MatTek Corporation), and then either Su9–GFP, DJ-1(WT), DJ-1(E18K), or DJ-1(E18H) plasmids were transfected using Fugene 6 (Promega). The resulting cells were incubated for another 24 h and then subjected to immunocytochemical analysis. For statistical analyses, cells were categorized based on the degree of DJ-1 or Su9–GFP mitochondrial colocalization. These analyses were carried out using 100 cells per siRNA condition. Error bars represent the mean±s.d. of three independent experiments.

### Accessible surface area determination

To determine the ASA value for each residue in the DJ-1 mitochondria localized mutants, we used the methods described in Ahmad et al. (2004). The DJ-1 crystal structure (PDB 1P5F) was used as a template for value determination.

### Recombinant protein purification

To obtain recombinant DJ-1 proteins from *E. coli*, WT and mutant DJ-1 genes were sub-cloned into pET21a(+) and pET28a(+) plasmids (Novagen - Merck Millipore), and then transformed into the *E. coli* strain BL21(DE3)+RIL (Agilent Technologies). His6-tagged WT DJ-1 and various DJ-1 mutants were purified by standard procedures using nickel-agarose (Ni-NTA Agarose, Qiagen) and an elution buffer (200 mM NaCl, 10 mM 2-mercaptoethanol, and 500–750 mM imidazole in 20 mM sodium phosphate buffer, pH 7.0). Recombinant DJ-1 proteins were dialyzed using 20 mM sodium phosphate buffer (pH 7.0) supplemented with 200 mM NaCl with or without 10% glycerol, and stored at −80°C. The WT and mutant DJ-1 were subjected to SDS-PAGE followed by Coomassie Brilliant Blue staining to confirm that the purified proteins resolved as single bands.

Recombinant chimera proteins (DJ17–GFP and DJ17–SNCA) were prepared as follows. *Escherichia coli* BL21-CodonPlus(DE3)-RIL competent cells (Agilent Technologies) transformed with pGEX-6P-1 plasmid encoding GST–DJ17–GFP or GST–DJ17–SNCA were grown in LB medium supplemented with 100 µg/ml ampicillin and 25 µg/ml chloramphenicol at 37°C. Expression of GST–DJ17–GFP and GST–DJ17–SNCA were induced by addition of 200 µM IPTG for 16 h at 18°C. The bacterial cell pellets after centrifugation (8000 ***g*** for 10 min) were resuspended in TBS buffer (50 mM Tris-HCl pH 7.5, 120 mM NaCl) supplemented with 50 µg/ml lysozyme (Wako), 1 µg/ml DNase I (Worthington Biochemical), 1 mM DTT (Roche), 1 mM MgCl_2_ (Wako), and protease inhibitor cocktail (Roche). The obtained cell suspension was sonicated (Advanced-Digital Sonifer, Branson), and insoluble proteins were removed by centrifugation (10,000 ***g*** for 15 min). The supernatants were mixed with equilibrated glutathione–Sepharose 4B (GE Healthcare) for 40 min at 4°C. The Sepharose was then loaded onto a column and washed with TBS buffer containing 1 mM Tris(2-carboxyethyl)phosphine (TCEP; Sigma). The bound chimera proteins (DJ17–GFP and DJ17–SNCA) were eluted from glutathione-Sepharose 4B with 1.5 ml TBS buffer containing 1 mM TECP and 20 units Prescission protease (GE Healthcare), and were subjected to the thermal shift analysis.

### Partial trypsin digestion

Recombinant DJ-1 (11 µM) was incubated with Trypsin Gold (1 µg/ml) in 200 mM Tris-HCl (pH 8.0) buffer solution at 37°C for 22 h. Samples were then mixed with 2× protease inhibitor and loading buffer, followed by denaturation at 98°C for 10 min. Samples were separated in a 12% acrylamide gel, followed by fixation and Coomassie Brilliant Blue staining. The bands were digitized with an ImageQuant LAS LAS4000 (GE Healthcare) and band intensity was determined using Image J software.

### Thermal shift assay

Determination of thermal stability of recombinant protein was undertaken using Protein Thermal Shift Dye (Thermo Fisher Scientific). DJ-1 and chimeric proteins (30 µM) were incubated in the reaction buffer containing 0.01% thermal shift dye according to the manufacturer's protocol. Fluorescence was measured using the ROX channel of a BioRad CFX96 Real Time PCR machine, with a 0.5°C/15 s per step (20–95°C) melting curve. To measure isatin-mediated stabilization, 500 µM of isatin (Tokyo Chemical Industry Co.) was added after all the other reagents were mixed. Peaks were determined using BioRad CFX Manager software.

### Isatin treatment in cells

To test whether isatin affected the subcellular localization of DJ-1, HeLa DJ-1-knockout cells were transiently transfected with plasmids expressing DJ-1 E18A or M26I mutants. After 4 h, 500 µM isatin (Tokyo Chemical Industry Co.) was added to media. After 16 h, the cells were fixed and subjected to immunocytochemistry as described above.

### Immunoblot analysis

Cell lysates were collected in TNE-N+ buffer (150 mM NaCl, 20 mM Tris-HCl, pH 8.0, 1 mM EDTA, and 1% NP-40) in the presence of various inhibitors. To detect the indicated proteins, anti-DJ-1 antibody 3E8 (MBL, 1:1000), anti-Tubulin antibody YL1/2 (Abcam, 1:3000), anti-HA antibody TANA2 (MBL, 1:2000), anti-TOMM40 antibody (a gift from Dr Toshihiko Oka, Department of Life Science, Rikkyo University, Japan, 1:1000), anti-TIMM23 antibody (BD Biosciences, 1:500), anti-TOMM20 antibody (Proteintech, 1:4000), anti-OPA1 antibody (BD Biosciences, 1:1000), anti-PGAM5 antibody (Abcam, 1:1000), anti-ATF4 antibody (CST, 1:500), anti-Actin antibody C4 (Merck Millipore, 1:2000) or an anti-GFP antibody ab6556 (Abcam, 1:2000) were used as primary antibodies, and HRP-conjugated goat anti-mouse IgG or anti-rabbit IgG antibody was used as secondary antibodies (Jackson ImmunoResearch). The HRP substrate consisted of the Western Lightning Plus-ECL (PerkinElmer) and images were captured on an ImageQuant LAS4000 (GE Healthcare). Quantification was performed using Image J software. Antibodies used in this study and their Research Resource Identifiers (RRIDs) are listed in Table S1. To provide cellular stress in Fig. S2, WT HeLa cells were treated with DMSO (NT, non-treated), 10 µM valinomycin (val) or 300 nM thapsigargin (TG) for 3 h, and then total cell lysates were prepared for immunoblotting.

### Doxycycline-induced chase

A pTRE-IRES plasmid (Takara Bio USA) was used to simultaneously express DJ-1 and EGFP, which are separated by the IRES, following the addition of doxycycline. Cells were transfected with the same amount of pTET3G (Takara Bio USA) and the appropriate pTRE-IRES plasmids. After 24 h, medium containing 50 ng/ml doxycycline (Dox) was added to initiate expression of EGFP and DJ-1 for 24 h. Cells were then washed and re-plated in medium without doxycycline. This time point was considered day 0, and the time was counted forward as day +1 and +2. To assess the degradation process of DJ-1 in detail, cells were treated with 10 µM CCCP, 10 µM valinomycin, and/or 2 µM epoxomicin following Dox-mediated induction.

### Statistical analysis

For quantitative colocalization and statistical analysis, the colocalization of TOMM20 with various DJ-1 mutants or chimera proteins (e.g. DJ17–GFP and DJ17–SNCA) was calculated using Zen software (Carl Zeiss) as a Pearson correlation coefficient. In the associated box-plots of the data, the dots indicate individual Pearson correlation coefficient data points for each cell, the center lines indicate the medians, the box limits indicate the 25th and 75th percentiles as determined by the R software package, and whiskers extend to 1.5 times the interquartile range from the 25th and 75th percentiles. Outliers are represented by dots outside of the whiskers, mean values are shown on the boxes. Statistical significance was calculated using one-way ANOVA in GraphPad Prism 6. For one-way ANOVA experiments, the Sidak correction for multiple comparisons was used. Differences were considered significant at *P*<0.01.

## Supplementary Material

Supplementary information
