# Supplementary material for: Unfolding is the driving force for mitochondrial import and degradation of the Parkinson's disease-related protein DJ-1
Source: J Cell Sci. 2021 Nov 25;134(22):jcs258653. doi: 10.1242/jcs.258653 (PMC8645234; doi:10.1242/jcs.258653)
Supplement: Supplementary information [file joces-134-258653-s1.pdf]

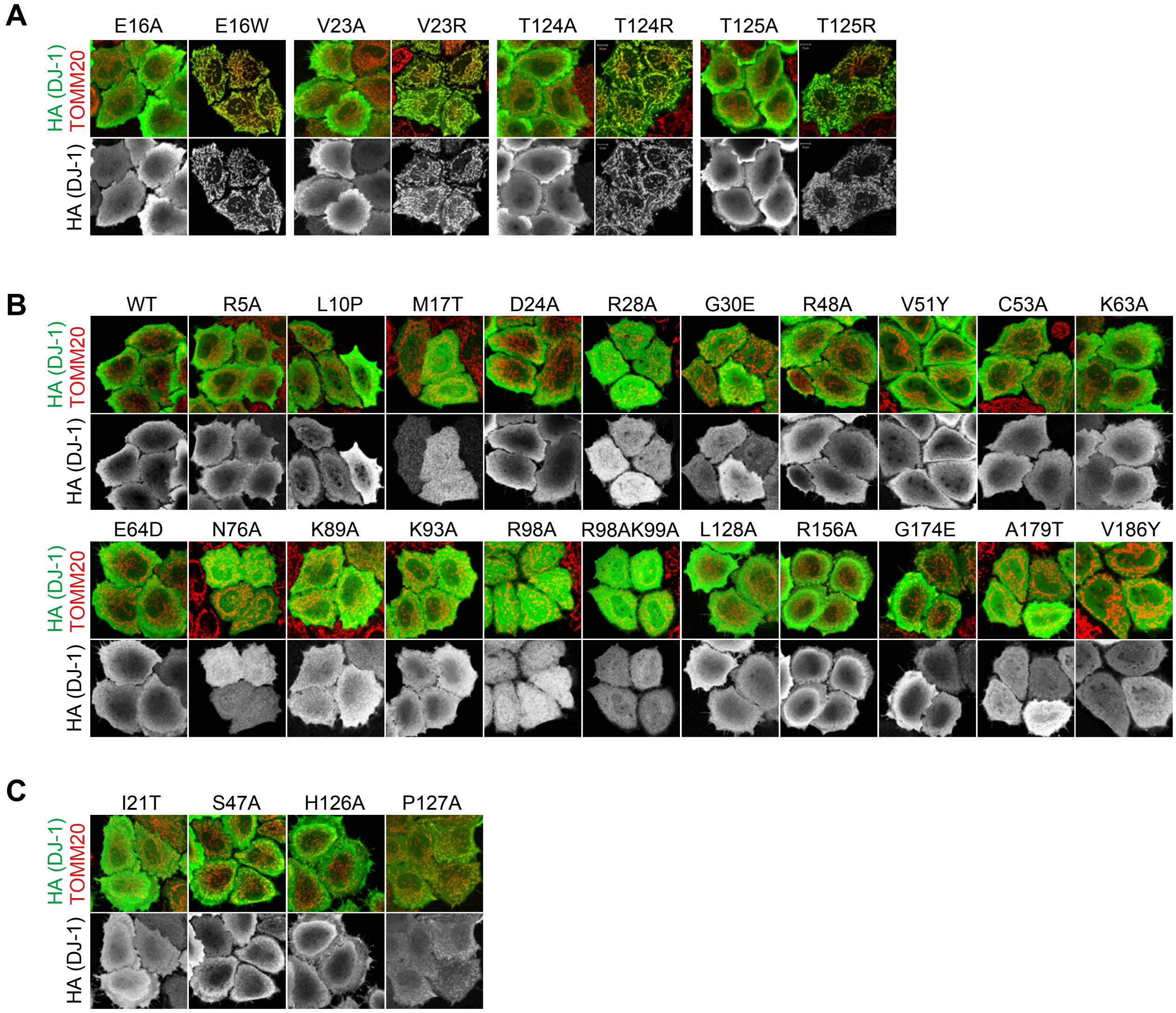

Fig. S1. (A) Representative images depicting the cytosolic localization of DJ-1 E16A, V23A, T124A, and T125A mutants with the corresponding mitochondrial localization of DJ-1 E16W, V23R, T124R, and T125R mutants.(B) Immunostaining images of DJ-1 mutants categorized as “cytosolic localization” in the quantitative analysis shown in Fig. 1D. (C) Immunostaining images of DJ-1 mutants categorized as “variable or mixed localization” in the quantitative analysis shown in Fig. 1D. In (A), (B), and (C), the top panels show localization of the DJ-1 constructs alone, and the bottom panels show merged images for the DJ-1 constructs (green) and the mitochondrial marker TOMM20 (red). All constructs were expressed in DJ-1 knockout cells.

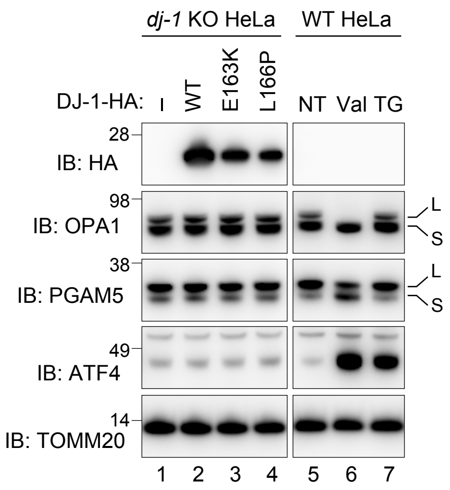

Fig. S2. No changes in the PGAM5 and OPA1 cleavage profile or the induction of ATF4 were observed following the expression of the mitochondria-localized pathogenic DJ-1 mutants E163K (lane 3) or L166P (lane 4). In contrast, when cells were treated with 10 µM Valinomycin for 3 hours (lane 6), changes to PGAM5 and Opa1 cleavage and ATF4 induction were observed. NT, cells were treated with DMSO (non-treated); Val, 10 µM valinomycin for 3 hours; TG, 300 nM thapsigargin for 3 hours.

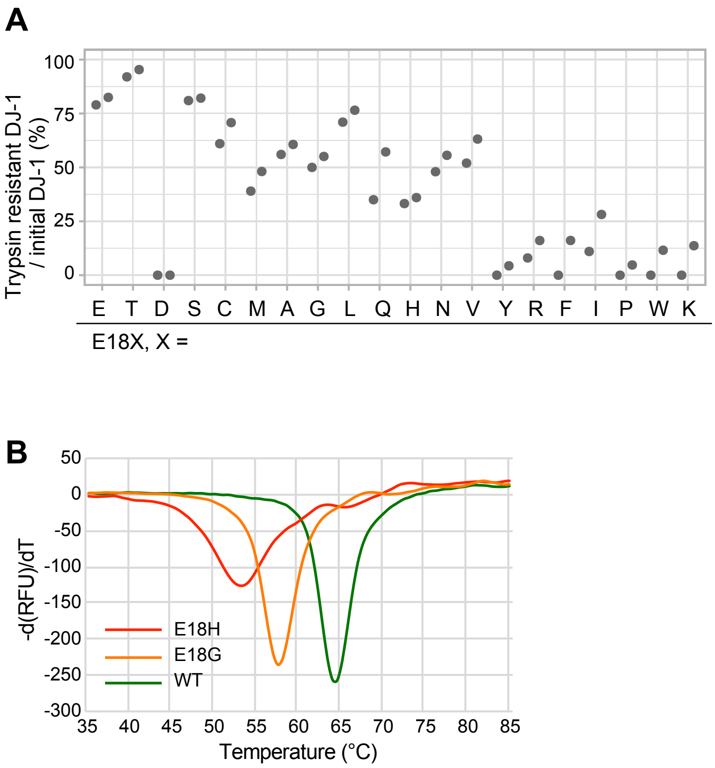

Fig. S3. (A) Recombinant C-terminal 6xHis-tagged E18X mutants were incubated with trypsin for 22 h, and the degree of trypsin sensitivity was quantified in two biological replicates. (B) Representative thermal spectra of the C-terminal 6xHis-tagged WT, E18G, and E18H DJ-1 proteins.

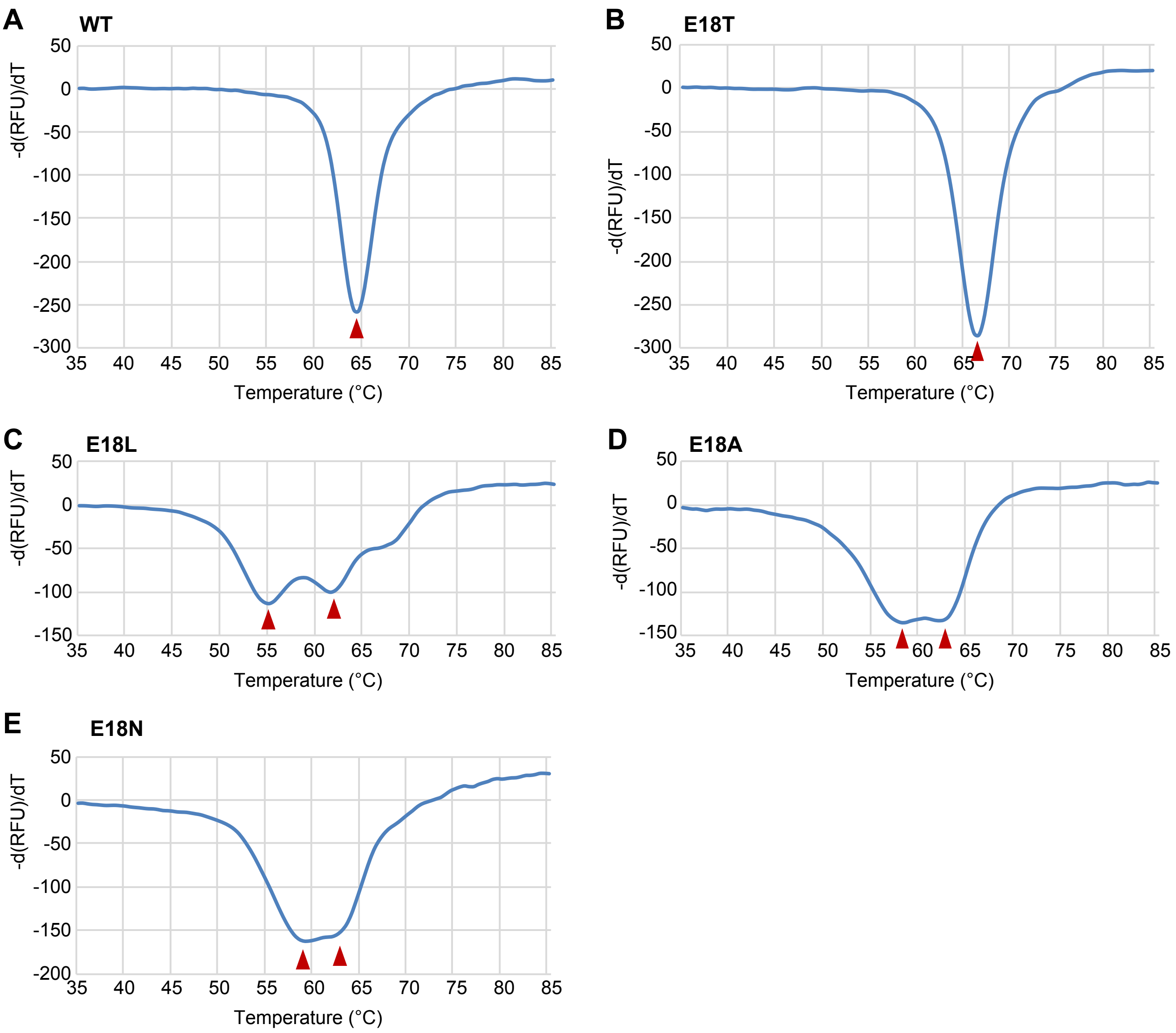

Fig. S4. Representative double-peak thermal spectra for the E18L/A/N mutants. Like WT (A), most spectra for the E18X mutants presented as a single peak for the E18T mutant (B). However, spectra for the E18L (C), E18A (D), and E18N (E) mutants had two peaks, suggesting unfolding transitions that are bimodal.

Journal of Cell Science • Supplementary information

Table S1.

REAGENT or RESOURCE	SOURCE	IDENTIFIER
Antibodies		
Mouse monoclonal anti-HA (TANA2)	MBL	Cat. # M180-3, RRID:AB_10951811
Rabbit polyclonal anti-TOMM20 (FL-145)	Santa Cruz Biotechnologies	Cat. # sc-11415, RRID:AB_2207533
Rabbit polyclonal anti-TOMM20 (11802-1-AP)	Proteintech	Cat# 11802-1-AP, RRID:AB_2207530
Mouse monoclonal anti-DJ-1 (3E8)	MBL	Cat# M043-3S, RRID:AB_592469
Mouse monoclonal anti-Actin (C4)	Merck Millipore	Cat. # MAB1501R, RRID:AB_2223041
Mouse monoclonal anti-DDDDK (FLA-1)	MBL	Cat. # M185-3L, RRID:AB_11123930
Rabbit polyclonal anti-GFP (ab6556)	Abcam	Cat# ab6556, RRID:AB_305564
Rabbit polyclonal anti-PGAM5 (ab126534)	Abcam	Cat# ab126534, RRID:AB_11127076
Rabbit anti-ATF4	Cell Signaling Technology	Cat# 11815, RRID:AB_2616025
Rat monoclonal anti-Tubulin (YL1/2)	Abcam	Cat# ab6160, RRID:AB_305328
Mouse monoclonal anti-TIMM23	BD Biosciences	Cat# 611223, RRID:AB_398755
Mouse monoclonal anti-OPA1	BD Biosciences	Cat# 612606, RRID:AB_399888
Goat polyclonal anti-HSP60 (N-20)	Santa Cruz Biotechnologies	Cat. # sc-1052, RRID:AB_631683
Goat anti-Rabbit IgG Alexa Fluor 488 conjugated	Thermo Fisher Scientific	Cat# A-11034, RRID:AB_2576217
Goat anti-Rabbit IgG Alexa Fluor 568 conjugated	Thermo Fisher Scientific	Cat# A-11036, RRID:AB_10563566
Goat anti-Mouse IgG Alexa Fluor 488 conjugated	Thermo Fisher Scientific	Cat# A-11029, RRID:AB_2534088
Goat anti-Mouse IgG Alexa Fluor 568 conjugated	Thermo Fisher Scientific	Cat# A-11031, RRID:AB_144696
Goat anti-Mouse IgG Alexa Fluor 647 conjugated	Thermo Fisher Scientific	Cat# A-21236, RRID:AB_2535805
Donkey anti-Goat IgG Alexa Fluor 488 conjugated	Abcam	Cat# ab150129, RRID:AB_2687506

Journal of Cell Science • Supplementary information

Donkey anti-Rabbit IgG Alexa Fluor 568 conjugated	Abcam	Cat# ab175470, RRID:AB_2783823
Goat Anti-Rabbit IgG horseradish peroxidase-linked	Jackson Immuno Research	Cat# 111-035-144, RRID:AB_2307391
Goat Anti-Mouse IgG horseradish peroxidase-linked	Jackson Immuno Research	Cat# 115-035-003, RRID:AB_10015289

Journal of Cell Science • Supplementary information

Table S2. List of plasmids used in this study. WT means wild type.

Plasmid name	Description	Source
pNMDJ1-1	For expression of DJ1-WT-HA	Kojima et al. 2016
pNMDJ1-2	For expression of DJ1-E18A-HA	Kojima et al. 2016
pNMDJ1-3	For expression of DJ1-C46S-HA	Kojima et al. 2016
pNMDJ1-4	For expression of DJ1-C53A-HA	Kojima et al. 2016
pNMDJ1-6	For expression of DJ1-L166P-HA	Kojima et al. 2016
pNMDJ1-7	For expression of DJ1-M26I-HA	Kojima et al. 2016
pNMDJ1-16	For expression of 6His-DJ1-WT	Matsuda et al. 2017
pNMDJ1-17	For expression of 6His-DJ1-L166P	Matsuda et al. 2017
pNMDJ1-18	For expression of 6His-DJ1-M26I	Matsuda et al. 2017
pNMDJ1-19	For expression of 6His-DJ1-E18A	Matsuda et al. 2017
pNMDJ1-32	For expression of DJ1-L10P-HA	Kojima et al. 2016
pNMDJ1-33	For expression of DJ1-E163K-HA	Kojima et al. 2016
pNMDJ1-35	For expression of DJ1-E64D-HA	Kojima et al. 2016
pNMDJ1-36	For expression of DJ1-A179T-HA	Kojima et al. 2016
pNMDJ1-37	For expression of 6His-DJ1-L10P	Matsuda et al. 2017
pNMDJ1-38	For expression of 6His-DJ1-E163K	Matsuda et al. 2017
pNMDJ1-40	For expression of 6His-DJ1-E64D	Matsuda et al. 2017
pNMDJ1-45	For expression of DJ1-H126A-HA	Kojima et al. 2016
pNMDJ1-48	For expression of 6His-DJ1-N76D	Matsuda et al. 2017
pNMDJ1-73	For expression of DJ1-C106K-HA	This study
pNMDJ1-84	For expression of DJ1-M17T-HA	This study
pNMDJ1-85	For expression of DJ1-I21T-HA	This study
pNMDJ1-86	For expression of DJ1-D24A-HA	This study
pNMDJ1-87	For expression of DJ1-R27A-HA	This study
pNMDJ1-88	For expression of DJ1-R28A-HA	This study
pNMDJ1-90	For expression of DJ1-V50Y-HA	This study
pNMDJ1-91	For expression of DJ1-V51Y-HA	This study
pNMDJ1-92	For expression of DJ1-K63A-HA	This study
pNMDJ1-93	For expression of DJ1-D68A-HA	This study
pNMDJ1-94	For expression of DJ1-R156A-HA	This study
pNMDJ1-95	For expression of DJ1-V186Y-HA	This study
pNMDJ1-103	For expression of DJ1-R5A-HA	This study
pNMDJ1-106	For expression of DJ1-E15A-HA	This study
pNMDJ1-107	For expression of DJ1-E16A-HA	This study
pNMDJ1-108	For expression of DJ1-V23A-HA	This study
pNMDJ1-109	For expression of DJ1-V33A-HA	This study

pNMDJ1-111	For expression of DJ1-N76A-HA	This study
pNMDJ1-112	For expression of DJ1-I105A-HA	This study
pNMDJ1-114	For expression of DJ1-G157E-HA	This study
pNMDJ1-115	For expression of DJ1-T124A-HA	This study
pNMDJ1-116	For expression of DJ1-T125A-HA	This study
pNMDJ1-118	For expression of DJ1-P127A-HA	This study
pNMDJ1-133	For expression of DJ1-K89A-HA	This study
pNMDJ1-134	For expression of DJ1-R98A-HA	This study
pNMDJ1-136	For expression of DJ1-R98A/K99A-HA	This study
pNMDJ1-137	For expression of DJ1-R48A-HA	This study
pNMDJ1-138	For expression of DJ1-G174E-HA	This study
pNMDJ1-141	For expression of DJ1-T34A-HA	This study
pNMDJ1-142	For expression of DJ1-G30E-HA	This study
pNMDJ1-146	For expression of DJ1-L128A-HA	This study
pNMDJ1-190	For expression of DJ1-T124R-HA	This study
pNMDJ1-191	For expression of DJ1-T125R-HA	This study
pNMDJ1-192	For expression of DJ1-A104R-HA	This study
pNMDJ1-194	For expression of DJ1-A14W-HA	This study
pNMDJ1-195	For expression of DJ1-E16W-HA	This study
pNMDJ1-200	For expression of DJ1-V23R-HA	This study
pNMDJ1-201	For expression of DJ1-V44P-HA	This study
pNMDJ1-202	For expression of DJ1-S47A-HA	This study
pNMDJ1-203	For expression of DJ1-K93A-HA	This study
pNMDJ1-204	For expression of DJ1-F164S-HA	This study
pNMDJ1-205	For expression of DJ1-E18T-HA	This study
pNMDJ1-206	For expression of DJ1-E18D-HA	This study
pNMDJ1-207	For expression of DJ1-E18S-HA	This study
pNMDJ1-208	For expression of DJ1-E18C-HA	This study
pNMDJ1-209	For expression of DJ1-E18K-HA	This study
pNMDJ1-210	For expression of DJ1-E18M-HA	This study
pNMDJ1-211	For expression of DJ1-E18G-HA	This study
pNMDJ1-212	For expression of DJ1-E18L-HA	This study
pNMDJ1-213	For expression of DJ1-E18Q-HA	This study
pNMDJ1-214	For expression of DJ1-E18H-HA	This study
pNMDJ1-215	For expression of DJ1-E18N-HA	This study
pNMDJ1-216	For expression of DJ1-E18V-HA	This study
pNMDJ1-217	For expression of DJ1-E18Y-HA	This study
pNMDJ1-218	For expression of DJ1-E18R-HA	This study

Journal of Cell Science • Supplementary information

pNMDJ1-219	For expression of DJ1-E18F-HA	This study
pNMDJ1-220	For expression of DJ1-E18I-HA	This study
pNMDJ1-221	For expression of DJ1-E18P-HA	This study
pNMDJ1-222	For expression of DJ1-E18W-HA	This study
pNMDJ1-223	For simultaneous expression of GFP and DJ1-WT-HA	This study
pNMDJ1-224	For simultaneous expression of GFP and DJ1-E18K-HA	This study
pNMDJ1-225	For simultaneous expression of GFP and DJ1-M26I-HA	This study
pNMDJ1-226	For expression of DJ1-E18K/R5P-HA	This study
pNMDJ1-227	For expression of DJ1-E18K/L7P-HA	This study
pNMDJ1-228	For expression of DJ1-E18K/L10P-HA	This study
pNMDJ1-229	For expression of DJ1-E18K/K12P-HA	This study
pNMDJ1-230	For expression of DJ1-E18K/V33P-HA	This study
pNMDJ1-231	For expression of DJ1-E18K/T34P-HA	This study
pNMDJ1-232	For expression of 6His-tagged DJ1-E18T	This study
pNMDJ1-233	For expression of 6His-tagged DJ1-E18D	This study
pNMDJ1-234	For expression of 6His-tagged DJ1-E18S	This study
pNMDJ1-235	For expression of 6His-tagged DJ1-E18C	This study
pNMDJ1-236	For expression of 6His-tagged DJ1-E18K	This study
pNMDJ1-237	For expression of 6His-tagged DJ1-E18M	This study
pNMDJ1-238	For expression of 6His-tagged DJ1-E18G	This study
pNMDJ1-239	For expression of 6His-tagged DJ1-E18L	This study
pNMDJ1-240	For expression of 6His-tagged DJ1-E18Q	This study
pNMDJ1-241	For expression of 6His-tagged DJ1-E18H	This study
pNMDJ1-242	For expression of 6His-tagged DJ1-E18N	This study
pNMDJ1-243	For expression of 6His-tagged DJ1-E18V	This study
pNMDJ1-244	For expression of 6His-tagged DJ1-E18Y	This study
pNMDJ1-245	For expression of 6His-tagged DJ1-E18R	This study
pNMDJ1-246	For expression of 6His-tagged DJ1-E18F	This study
pNMDJ1-247	For expression of 6His-tagged DJ1-E18I	This study
pNMDJ1-248	For expression of 6His-tagged DJ1-E18P	This study
pNMDJ1-249	For expression of 6His-tagged DJ1-E18W	This study
pNMDJ1-250	For expression of DJ1-deltaC (1-134)-HA	This study
pNMDJ1-253	For expression of DJ64/YajL-deltaC-HA	This study
pNMDJ1-254	For expression of YajL-HA	This study
pNMDJ1-255	For expression of YajL-deltaC (1-134)-HA	This study
pNMDJ1-258	For expression of YajL63/DJ1-deltaC-HA	This study
pNMDJ1-259	For expression of DJ17-GFP	This study
pNMDJ1-260	For expression of DJ43-GFP	This study

Journal of Cell Science • Supplementary information

pNMDJ1-262	For expression of SNCA-HA	This study
pNMDJ1-263	For expression of DJ17- SNCA-HA	This study
pNMDJ1-264	For expression of DJ43- SNCA-HA	This study
pNMDJ1-266	For expression of GST-DJ17-GFP	This study
pNMDJ1-267	For expression of GST-DJ17-SNCA	This study
pCMV-Tet3G	For Dox inducible expression	Takara Bio (Japan)
pTRE3G	For Dox inducible expression	Takara Bio (Japan)
pSu9-GFP	For expression of Su9(MTS)-GFP	Ishihara et al. 2006, Okatsu et al. 2015

References:
Ishihara *et al*. 2006, EMBO J. 25, 2966-2977. doi:10.1038/sj.emboj.7601184
Okatsu *et al*. 2015, J. Cell Sci. 128, 964-978. doi:10.1242/jcs.161000a
Kojima *et al*. 2016, Genes Cells 21, 772-788. doi:10.1111/gtc.12382
Matsuda *et al*. 2017, Sci Rep. 7, 12816. doi:10.1038/s41598-017-13146-0.
